# Incidence and risk factors of ischemic stroke in patients with cancer-associated venous thromboembolism: from the Contemporary Management and Outcomes in Patients With Venous Thromboembolism Registry-2

**DOI:** 10.1016/j.rpth.2024.102617

**Published:** 2024-10-30

**Authors:** Toru Sato, Yoshito Ogihara, Yugo Yamashita, Takeshi Morimoto, Ryuki Chatani, Kazuhisa Kaneda, Yuji Nishimoto, Nobutaka Ikeda, Yohei Kobayashi, Satoshi Ikeda, Kitae Kim, Moriaki Inoko, Toru Takase, Shuhei Tsuji, Maki Oi, Takuma Takada, Kazunori Otsui, Jiro Sakamoto, Takeshi Inoue, Shunsuke Usami, Po-Min Chen, Kiyonori Togi, Norimichi Koitabashi, Seiichi Hiramori, Kosuke Doi, Hiroshi Mabuchi, Yoshiaki Tsuyuki, Koichiro Murata, Kensuke Takabayashi, Hisato Nakai, Daisuke Sueta, Wataru Shioyama, Tomohiro Dohke, Ryusuke Nishikawa, Takeshi Kimura, Kaoru Dohi, Contemporary Management and Outcomes in Patients With Venous Thromboembolism Registry-2 Investigators

**Affiliations:** 1 Department of Cardiology and Nephrology, Mie University Graduate School of Medicine, Tsu, Japan; 2 Department of Cardiovascular Medicine, Graduate School of Medicine, Kyoto University, Kyoto, Japan; 3 Department of Clinical Epidemiology, Hyogo College of Medicine, Nishinomiya, Japan; 4 Department of Cardiovascular Medicine, Kurashiki Central Hospital, Kurashiki, Japan; 5 Department of Cardiology, Hyogo Prefectural Amagasaki General Medical Center, Amagasaki, Japan; 6 Division of Cardiovascular Medicine, Toho University Ohashi Medical Center, Tokyo, Japan; 7 Department of Cardiovascular Center, Osaka Red Cross Hospital, Osaka, Japan; 8 Department of Cardiovascular Medicine, Nagasaki University Graduate School of Biomedical Sciences, Nagasaki, Japan; 9 Department of Cardiovascular Medicine, Kobe City Medical Center General Hospital, Kobe, Japan; 10 Cardiovascular Center, The Tazuke Kofukai Medical Research Institute, Kitano Hospital, Osaka, Japan; 11 Department of Cardiology, Kinki University Hospital, Osaka, Japan; 12 Department of Cardiology, Japanese Red Cross Wakayama Medical Center, Wakayama, Japan; 13 Department of Cardiology, Japanese Red Cross Otsu Hospital, Otsu, Japan; 14 Department of Cardiology, Tokyo Women’s Medical University, Tokyo, Japan; 15 Department of General Internal Medicine, Kobe University Hospital, Kobe, Japan; 16 Department of Cardiology, Tenri Hospital, Tenri, Japan; 17 Department of Cardiology, Shiga General Hospital, Moriyama, Japan; 18 Department of Cardiology, Kansai Electric Power Hospital, Osaka, Japan; 19 Department of Cardiology, Osaka Saiseikai Noe Hospital, Osaka, Japan; 20 Division of Cardiology, Nara Hospital, Kinki University Faculty of Medicine, Ikoma, Japan; 21 Department of Cardiovascular Medicine, Gunma University Graduate School of Medicine, Maebashi, Japan; 22 Department of Cardiology, Kokura Memorial Hospital, Kokura, Japan; 23 Department of Cardiology, National Hospital Organization Kyoto Medical Center, Kyoto, Japan; 24 Department of Cardiology, Koto Memorial Hospital, Higashiomi, Japan; 25 Division of Cardiology, Shimada General Medical Center, Shimada, Japan; 26 Department of Cardiology, Shizuoka City Shizuoka Hospital, Shizuoka, Japan; 27 Department of Cardiology, Hirakata Kohsai Hospital, Hirakata, Japan; 28 Department of Cardiovascular Medicine, Sugita Genpaku Memorial Obama Municipal Hospital, Obama, Japan; 29 Department of Cardiovascular Medicine, Graduate School of Medical Sciences, Kumamoto University, Kumamoto, Japan; 30 Department of Cardiovascular Medicine, Shiga University of Medical Science, Otsu, Japan; 31 Division of Cardiology, Kohka Public Hospital, Koka, Japan

**Keywords:** cancer, direct oral anticoagulants, ischemic stroke, venous thromboembolism, risk factors

## Abstract

**Background:**

Ischemic stroke is a serious complication in patients with cancer-associated venous thromboembolism (CAVTE), although data remain scarce in the direct oral anticoagulant era.

**Objectives:**

This study aimed to investigate the incidence and identify predictive risk factors of ischemic stroke in patients with CAVTE.

**Methods:**

From the Contemporary Management and Outcomes in Patients With Venous Thromboembolism Registry-2 enrolling 5197 venous thromboembolism (VTE) patients across 31 centers in Japan between January 2015 and August 2020, we selected 1507 patients with active cancer. We calculated the cumulative incidence function of ischemic stroke accounting for the competing risk of death and investigated risk factors for ischemic stroke in a subdistribution hazard model of multivariable analysis.

**Results:**

During a median follow-up period of 1020 days, 71 patients (4.7%) developed ischemic stroke, and the cumulative incidence of ischemic stroke was 4.0% at 1 year and 4.7% at 3 years. Independent risk factors of ischemic stroke included pancreatic cancer (hazard ratio [HR], 4.24; 95% CI, 2.13-8.43), ovarian cancer (HR, 2.82; 95% CI, 1.31-6.08), lung cancer (HR, 2.35; 95% CI, 1.20-4.57), dyslipidemia (HR, 1.76; 95% CI, 1.01-3.09), metastasis (HR, 1.70; 95% CI, 1.02-2.82), higher D-dimer at VTE diagnosis (HR, 1.09; 95% CI, 1.04-1.14), and younger age (HR, 0.84; 95% CI, 0.71-0.999).

**Conclusion:**

In this large VTE registry in the direct oral anticoagulant era, the cumulative incidence of ischemic stroke was 4.0% at 1 year and 4.7% at 3 years in patients with CAVTE, and several independent risk factors of ischemic stroke were identified, including pancreatic cancer, ovarian cancer, lung cancer, dyslipidemia, metastasis, higher D-dimer at VTE diagnosis, and younger age.

## Essentials

• Ischemic stroke is a serious complication in cancer-associated venous thromboembolism.
• Its incidence and risk factors in the era of direct oral anticoagulants remain understudied.
• The cumulative 3-year incidence of ischemic stroke was 4.7% in patients with cancer-associated venous thromboembolism.
• Pancreatic, ovarian, and lung cancers, dyslipidemia, metastasis, high D-dimer, and young age were identified as risk factors for ischemic stroke.

## 1 Introduction

Patients with cancer-associated venous thromboembolism (CAVTE) sometimes show marked thrombogenesis, which could cause serious additional thrombotic complications such as ischemic stroke through a complex interplay of mechanisms [[1], [2], [3]]. These mechanisms include hypercoagulability caused by the malignancy itself, cardiogenic cerebral embolization resulting from nonbacterial thrombotic endocarditis, microthrombosis and embolization associated with disseminated intravascular coagulation syndrome, and hyperviscosity and hypoperfusion due to dehydration [1,2,4]. Ischemic stroke can lead to a markedly decreased quality of life, interruption of essential cancer treatments, and mortality [3,5,6], underscoring the importance of adopting appropriate management strategies in patients with CAVTE.

Recent randomized clinical trials revealed the efficacy and safety of direct oral anticoagulants (DOACs) for patients with CAVTE compared with low-molecular-weight heparin [[7], [8], [9], [10]], and the current guidelines recommend DOACs as a first-line treatment option for CAVTE [[11], [12], [13], [14], [15]]. However, there may be some concerns regarding the use of DOACs for CAVTE patients with marked thrombogenesis, which is sometimes referred as Trousseau syndrome. However, data on the issue have not been fully evaluated in the DOAC era. Therefore, the current study aimed to investigate the incidence and identify predictive risk factors of ischemic stroke in patients with CAVTE, using a large-scale observational venous thromboembolism (VTE) database in the DOAC era.

## 2 Methods

### 2.1 Study design and population

The Contemporary Management and Outcomes in Patients With Venous Thromboembolism (COMMAND VTE) Registry-2 is a physician-initiated, multicenter, retrospective cohort study, which enrolled consecutive patients with acute symptomatic VTE objectively, confirmed by imaging examination or autopsy, across 31 centers in Japan from January 2015 to August 2020, after the introduction of DOACs for VTE in Japan. The design of the study was previously reported in detail [16]. Briefly, we searched hospital databases for clinical diagnoses and/or imaging examinations and enrolled consecutive patients who met the definitions of acute symptomatic VTE diagnosed within 31 days from symptom onset during the study period [16]. The research protocol was approved by the relevant review boards or ethics committees at all 31 participating centers (Supplementary Appendix S1). Informed consent was obtained via an opt-out approach on each hospital’s website, due to the use of clinical information obtained during routine clinical practices. This study was conducted in accordance with the guidelines for epidemiological studies issued by the Ministry of Health, Labour, and Welfare in Japan.

**Figure 1:**
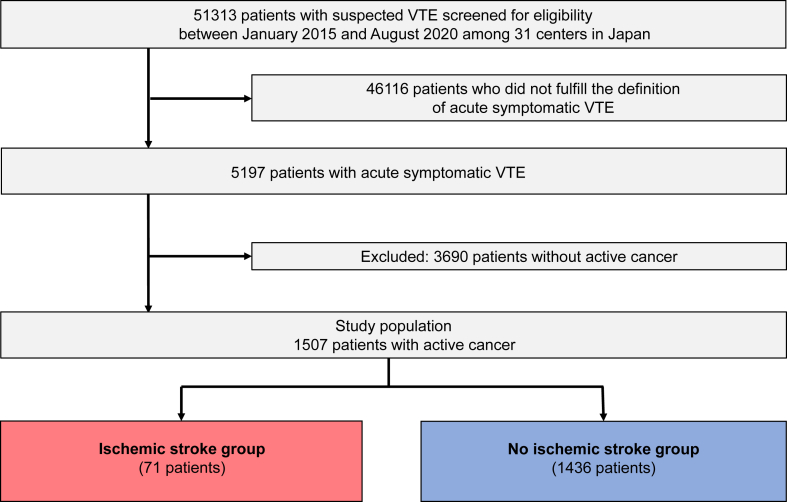
Study flowchart. Venous thromboembolism (VTE) included pulmonary embolism and/or deep vein thrombosis.

In the current study, after excluding 3690 patients without active cancer, we identified 1507 patients with active cancer, who were divided into 2 groups according to the development of ischemic stroke during the entire follow-up period: ischemic stroke group and no ischemic stroke group (Figure 1). We investigated the incidence of ischemic stroke after VTE diagnosis in patients with CAVTE during a median follow-up period of 1020 (IQR, 603-1666) days for surviving patients. We compared clinical characteristics and treatment strategies between the 2 groups and explored the risk factors for ischemic stroke.

### 2.2 Data collection and definitions

Baseline data were collected from hospital charts or databases according to the prespecified definitions, utilizing an electronic case report form in a web-based database system. The study investigators at each institution were responsible for data entry, with automatic checks for missing or contradictory input and values outside the expected range. Additional editing checks were performed at the general office of the registry. Body weight, body mass index, D-dimer, and leukocyte counts had missing values. Analyses were conducted using available data without imputation. Collection of follow-up information was mainly conducted through review of hospital charts, and additional follow-up information was collected through contact with patients, relatives, and/or referring physicians by phone and/or mail with questions regarding the vital status, clinical events, invasive procedure, and status of anticoagulation therapy. The independent clinical event committee (Supplementary Appendix S2), unaware of the patient characteristics, reviewed the detailed clinical course of patients and adjudicated the clinical events. In cases of inconsistencies, the final adjudication of clinical events was based on the full consensus of the independent clinical event committee.

Patients with active cancer were defined as those undergoing cancer treatment, such as chemotherapy or radiotherapy; those scheduled for cancer surgery; those with metastases to other organs; and/or those with terminal cancer (expected life expectancy of 6 months or less) at the time of VTE diagnosis [17]. This also included patients recently diagnosed with cancer who developed VTE postoperatively, as well as those diagnosed with VTE prior to the initiation of cancer treatments. In cases of multiple primary cancers, each cancer type was counted separately. For example, if a patient had both lung and large intestine cancers, the patient was considered to have 2 distinct risk factors. Ischemic stroke was diagnosed based on a neurologic disorder that developed suddenly and lasted for more than 24 hours, and confirmed based on obstruction of cerebrovascular blood flow by imaging examinations such as computed tomography, magnetic resonance imaging, and cerebral angiography. Detailed definitions of other patient characteristics are described in Supplementary Appendix S3.

Anticoagulation therapy cessation was classified as interruption or discontinuation of treatment according to the prespecified designations [16]. Briefly, treatment discontinuation was defined as withdrawal of anticoagulation therapy for more than 14 days for any reason, such as the treating physician’s decision in the absence of adverse events, bleeding events, drug side effects, and nonadherence.

### 2.3 Statistical analysis

Categorical variables are presented as numbers and percentages. Continuous variables are presented as mean and SD or median and IQR based on their distributions. Categorical variables were compared using the Fisher exact test. Continuous variables were compared using Student’s *t*-test or the Mann–Whitney-U test based on their distributions. The cumulative incidence function was used to estimate the cumulative incidence of ischemic stroke after VTE diagnosis, considering death as a competing risk. Additionally, we constructed a subdistribution hazard model to estimate the hazard ratios (HRs) and 95% CIs of the variables for the development of ischemic stroke, considering death as a competing risk. Due to the relatively small number of ischemic stroke events, we selected a limited set of 10 potential predictive risk factors of ischemic stroke during follow-up for the multivariate model, including age, sex, and variables with *P* values <0.10 in the univariate models among baseline characteristics (D-dimer, dyslipidemia, lung cancer, large intestine cancer, blood cancer, ovarian cancer, pancreatic cancer, and metastasis). Since the frequency of missing data for D-dimer was low at 6.8% (102/1507), and there were no missing data for the other factors included in the multivariable model, we did not perform data imputation. As a sensitivity analysis, we employed a stepwise method based on Akaike information criteria to refine the initial 10 covariates and then used the refined set of covariates in a subdistribution hazard model to reassess the relationships. All statistical analyses were performed using EZR (Saitama Medical Center, Jichi Medical University), which is a graphical user interface for R (version 2.13.0; The R Foundation for Statistical Computing). All reported *P* values were 2-tailed, and significance was set at a *P* value of <.05.

## 3 Results

### 3.1 Patient characteristics

In the entire study population, the median age was 70 (IQR, 61-77) years, with 56% of patients being women (Table 1). The mean body weight and body mass index were 56.6 ± 12.5 kg and 22.4 ± 4.2 kg/m^2^, respectively. Among the 1507 patients with CAVTE, 50% (756/1507) were censored during the 1-year follow-up period, with cancer-related death being the most common cause (68%). During the entire follow-up period, ischemic stroke occurred in 71 patients (4.7%; Figure 1).

**Table 1:** Baseline characteristics.

	Total (*N* = 1507)	Ischemic stroke (*n* = 71)	No ischemic stroke (*n* = 1436)	*P* value
Baseline demographics				
Age (y)	70 (61-77)	66 (59-73)	70 (61-77)	.02
Women	844 (56%)	48 (68%)	796 (55%)	.0498
Body weight (kg; *n* = 1484)	56.6 ± 12.5	55.5 ± 12.0	56.7 ± 12.6	.43
Body mass index (kg/m^2^; *n* = 1464)	22.4 ± 4.2	21.7 ± 3.9	22.5 ± 4.2	.14
Body mass index ≥ 30 kg/m^2^	74 (4.9%)	1 (1.4%)	73 (5.1%)	.26
Comorbidities				
Hypertension	575 (38%)	32 (45%)	543 (38%)	.26
Diabetes mellitus	237 (16%)	14 (20%)	223 (16%)	.32
Dyslipidemia	301 (20%)	20 (28%)	281 (20%)	.09
Chronic kidney disease	276 (18%)	10 (14%)	266 (19%)	.43
Chronic heart disease	112 (7.4%)	4 (5.6%)	108 (7.5%)	.82
Heart failure	32 (2.1%)	1 (1.4%)	31 (2.2%)	1.00
History of myocardial infarction	27 (1.8%)	0 (0%)	27 (1.8%)	.64
Atrial fibrillation	64 (4.2%)	3 (4.2%)	61 (4.2%)	1.00
Chronic lung disease	121 (8.0%)	7 (9.9%)	114 (7.9%)	.50
History of stroke	89 (5.9%)	6 (8.5%)	83 (5.8%)	.31
Liver cirrhosis	18 (1.2%)	1 (1.4%)	17 (1.2%)	.58
Autoimmune disorder	74 (4.9%)	3 (4.2%)	71 (4.9%)	1.00
Inflammatory bowel disease	6 (0.4%)	0 (0%)	6 (0.4%)	1.00
Antiphospholipid syndrome	9 (0.6%)	0 (0%)	9 (0.6%)	1.00
Varicose veins	43 (2.9%)	2 (2.8%)	41 (2.9%)	1.00
History of VTE	82 (5.4%)	4 (5.6%)	78 (5.4%)	.79
History of major bleeding	100 (6.6%)	6 (8.5%)	94 (6.5%)	.47
Presentation				
PE with or without DVT	771 (51%)	39 (55%)	732 (51%)	.54
Hypoxemia	311/771 (40%)	10/39 (26%)	301/732 (41%)	.07
Shock	54/771 (7.0%)	0/39 (0%)	54/732 (7.4%)	.10
Cardiac arrest/collapse	17/771 (2.2%)	0/39 (0%)	17/732 (2.3%)	1.00
DVT only	736 (49%)	32 (45%)	704 (49%)	.54
Proximal DVT in lower extremities	414/736 (56%)	17/32 (54%)	397/704 (56%)	.72
Laboratory tests at diagnosis				
Anemia	1163 (77%)	53 (75%)	1110 (77%)	.57
Leukocytes (/μL; *n* = 1505)	7110 (5040-9820)	7680 (4650-9560)	7100 (5060-9895)	.99
Thrombocytopenia	128 (8.5%)	8 (11%)	120 (8.4%)	.38
D-dimer (μg/mL; *n* = 1405)	10.3 (4.9-22.6)	25.2 (9.9-38.2)	10.1 (4.8-21.2)	<.001
Hereditary thrombophilia	26 (1.7%)	3 (4.2%)	23 (1.6%)	.12
Cancer site				
Lung	240 (16%)	17 (24%)	223 (16%)	.07
Blood	183 (12%)	3 (4.2%)	180 (13%)	.04
Large intestine	166 (11%)	2 (2.8%)	164 (11%)	.02
Uterus	132 (8.8%)	5 (7.0%)	127 (8.8%)	.83
Pancreas	130 (8.6%)	17 (24%)	113 (7.9%)	<.001
Ovary	120 (8.0%)	12 (17%)	108 (7.5%)	.01
Stomach	97 (6.4%)	4 (5.6%)	93 (6.5%)	1.00
Prostate	86 (5.7%)	2 (2.8%)	84 (5.8%)	.43
Kidney/urinary tract	64 (4.2%)	1 (1.4%)	63 (4.4%)	.36
Breast	61 (4.0%)	2 (2.8%)	59 (4.1%)	1.00
Gallbladder/bile duct	47 (3.1%)	4 (5.6%)	43 (3.0%)	.28
Bladder	46 (3.1%)	0 (0%)	46 (3.2%)	.17
Brain	34 (2.3%)	1 (1.4%)	33 (2.3%)	1.00
Esophagus	26 (1.7%)	0 (0%)	26 (1.8%)	.63
Liver	23 (1.5%)	0 (0%)	23 (1.6%)	.62
Thyroid gland	11 (0.7%)	0 (0%)	11 (0.8%)	1.00
Skin	9 (0.6%)	0 (0%)	9 (0.6%)	1.00
Others	95 (6.3%)	6 (8.5%)	89 (6.2%)	.45
Cancer status				
Terminal cancer	222 (15%)	7 (9.9%)	215 (15%)	.30
Metastasis	417 (28%)	32 (45%)	385 (27%)	.002
Under chemotherapy	765 (51%)	39 (55%)	726 (51%)	.54
Under radiotherapy	58 (3.8%)	3 (4.2%)	55 (3.8%)	.75
Scheduled for surgery	167 (11%)	7 (9.9%)	160 (11%)	.85
Others	254 (17%)	9 (13%)	245 (17%)	.42

Categorical variables are presented as the number and percentages. Continuous variables are presented as the mean and SD or median and IQR based on their distributions. Categorical variables were compared using the Fisher exact test. Continuous variables were compared using Student’s *t*-test or the Mann–Whitney-U test based on their distributions. Body weight, body mass index, D-dimer, and leukocyte counts had missing values. History of major bleeding was diagnosed if the patient had a history of International Society on Thrombosis and Hemostasis major bleeding. Hypoxemia was defined as arterial oxygen partial pressure of <60 mmHg or percentage saturation of hemoglobin with oxygen of <90%. Shock was defined as systolic blood pressure of <90 mmHg for at least 15 minutes, a pressure drop of ≥40 mmHg for at least 15 minutes, or requiring inotropic support. Anemia was defined as hemoglobin level of <13 g/dL for men and <12 g/dL for women. Thrombocytopenia was defined as platelet count of <100 × 10^9^/L. Hereditary thrombophilia included protein C deficiency, protein S deficiency, and antithrombin III deficiency. Other detailed definitions of patient characteristics are presented in Methods and Supplementary Appendix S3.

DVT, deep vein thrombosis; PE, pulmonary embolism; VTE, venous thromboembolism.

Patients in the ischemic stroke group were significantly younger and more often women compared with those in the no ischemic stroke group (median age, 66 vs 70 years; *P* = .02; 68% vs 55%; *P* = .0498, respectively). There was no significant difference in comorbidities between the 2 groups. D-dimer at VTE diagnosis was significantly higher in the ischemic stroke group than in the no ischemic stroke group (25.2 vs 10.1 μg/mL, respectively; *P* < .001). Regarding cancer sites, ovarian and pancreatic cancers were more common in the ischemic stroke group, while large intestine and blood cancers were less common in the ischemic stroke group (Table 1). Regarding the cancer status, metastasis was more common in the ischemic stroke group than in the no ischemic stroke group (45 vs 27%, respectively; *P* = .002). The median age was younger in patients with ovarian cancer than in those with other cancer types. Moreover, among patients with ovarian and lung cancers, the median age was younger in the ischemic stroke group than in the no ischemic stroke group (Supplementary Table S1).

### 3.2 Treatment strategies

There was no significant difference in initial parenteral therapy between the 2 groups (Table 2). The proportion of DOACs as oral anticoagulation therapy was 80% in the entire population and significantly higher in the ischemic stroke group than in the no ischemic stroke group (90% vs 79%, respectively; *P* = .02). Among the 71 patients in the ischemic stroke group, anticoagulation therapy was discontinued in 21 (30%) prior to the onset of ischemic stroke. Another 4 patients (5.6%) did not receive any anticoagulation therapy following VTE diagnosis. In all 4 cases, anemia was present and anticoagulation therapy for VTE was not initiated because of the associated bleeding risk. Among the 71 patients in the ischemic stroke group, 46 patients received anticoagulation therapy at the onset of ischemic stroke. Of these 46 patients, anticoagulation therapy was switched from DOACs to heparin in 4 patients after the onset of ischemic stroke.

**Table 2:** Treatment strategies.

	Total (*N* = 1507)	Ischemic stroke (*n* = 71)	No ischemic stroke (*n* = 1436)	*P* value
Initial parenteral therapy	722 (48%)	32 (45%)	690 (48%)	.72
Heparin	704 (47%)	32 (45%)	672 (47%)	.40
Single injection at diagnosis	34/704 (4.8%)	3/32 (9.4%)	31/672 (4.6%)	.20
Continuous injection	670/704 (95%)	29/32 (91%)	641/672 (95%)	
Fondaparinux	23 (1.5%)	0 (0%)	23 (1.6%)	.62
Thrombolysis	43 (2.9%)	0 (0%)	43 (3.0%)	.26
Inferior vena cava filter use	137 (9.1%)	8 (11%)	129 (9.0%)	.52
Ventilator support	19 (1.3%)	0 (0%)	19 (1.3%)	1.00
Percutaneous cardiopulmonary support	6 (0.4%)	0 (0%)	6 (0.4%)	1.00
Oral anticoagulation therapy	1363 (90%)	66 (93%)	1297 (90%)	.68
Vitamin K antagonist (warfarin)	164 (11%)	2 (2.8%)	162 (11%)	.02
DOAC	1199 (80%)	64 (90%)	1135 (79%)	
Dabigatran	2/1199 (0.2%)	0/64 (0%)	2/1135 (0.2%)	1.00
Rivaroxaban	297/1199 (25%)	15/64 (23%)	282/1135 (25%)	.88
Initial intensive treatment: 30 mg/d	210/297 (71%)	10/15 (67%)	200/282 (71%)	.77
Maintenance dose: 15 mg/d	249/297 (84%)	11/15 (73%)	238/282 (84%)	1.00
Maintenance dose: 10 mg/d	21/297 (7.1%)	1/15 (6.7%)	20/282 (7.1%)	
Apixaban	257/1199 (21%)	16/64 (25%)	241/1135 (21%)	.53
Initial intensive treatment: 20 mg/d	134/257 (52%)	9/16 (56%)	125/241 (52%)	.80
Maintenance dose: 10 mg/d	223/257 (87%)	15/16 (94%)	208/241 (86%)	.37
Maintenance dose: 5 mg/d	24/257 (9.3%)	0/16 (0%)	24/241 (10%)	
Edoxaban	643/1199 (54%)	33/64 (52%)	610/1135 (54%)	.80
60 mg/d	183/643 (29%)	9/33 (27%)	174/610 (29%)	1.00
30 mg/d	453/643 (71%)	24/33 (73%)	429/610 (70%)	
15 mg/d	7/643 (1.1%)	0/33 (0%)	7/610 (1.1%)	
Concomitant medications at discharge				
Corticosteroids	210 (14%)	13 (18%)	197 (14%)	.29
Nonsteroidal anti-inflammatory drugs	190 (13%)	9 (13%)	181 (13%)	1.00
Proton pump inhibitors/H_2_ blockers	763 (51%)	40 (56%)	723 (50%)	.33
Statins	190 (13%)	14 (20%)	176 (12%)	.07
Antiplatelet agents	85 (5.6%)	3 (4.2%)	82 (5.7%)	.79

Categorical variables are presented as numbers and percentages and compared using the Fisher exact test. Initial parenteral therapy included heparin (single or continuous injection), fondaparinux, and thrombolysis (urokinase or tissue plasminogen activator) within 10 days after the diagnosis. Antiplatelet drugs included aspirin, ticlopidine, clopidogrel, prasugrel, ticagrelor, and cilostazol.

DOAC, direct oral anticoagulant.

### 3.3 Clinical events

**Figure 2:**
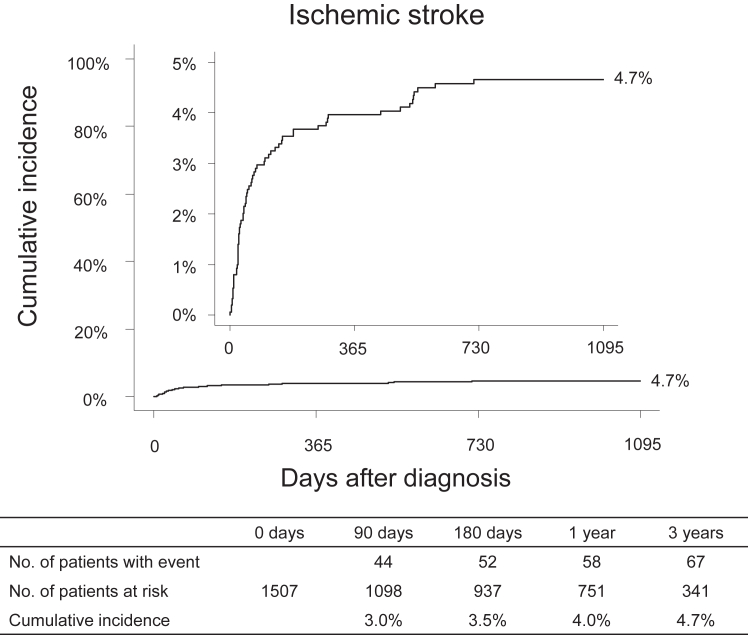
Cumulative incidence of ischemic stroke. The cumulative incidence function was used to estimate the cumulative incidence of ischemic stroke after venous thromboembolism diagnosis.

The cumulative incidence of ischemic stroke was 3.0% at 90 days, 4.0% at 1 year, and 4.7% at 3 years (Figure 2). The median time to the development of ischemic stroke after VTE diagnosis was 50 (IQR, 23-187) days. Furthermore, in the analysis comparing patients with and without metastasis, the cumulative incidence of ischemic stroke in patients with metastasis was 7% at 1 year and 7.9% at 3 years, which was higher than that in those without metastasis (Supplementary Figure S1).

Multivariate analysis revealed that the independent risk factors of ischemic stroke during follow-up included age (HR, 0.84; 95% CI, 0.71-0.999; *P* = .048), higher D-dimer at VTE diagnosis (HR, 1.09; 95% CI, 1.04-1.14; *P* < .001), dyslipidemia (HR, 1.76; 95% CI, 1.01-3.09; *P* = .048), pancreatic cancer (HR, 4.24; 95% CI, 2.13-8.43; *P* < .001), ovarian cancer (HR, 2.82; 95% CI, 1.31-6.08; *P* = .008), lung cancer (HR, 2.35; 95% CI, 1.20-4.57; *P* = .01), and metastasis (HR, 1.70; 95% CI, 1.02-2.82; *P* = .04; Table 3).

**Table 3:** Risk factors for the development of ischemic stroke by subdistribution hazard model.

Variables	Crude HR (95% CI)	*P* value	Adjusted HR (95% CI)	*P* value
Age (each 10 y)	0.86 (0.75-0.99)	.04	0.84 (0.71-0.999)	.048
Women	1.67 (1.02-2.74)	.04	1.39 (0.80-2.43)	.25
D-dimer (each 10 μg/mL)	1.07 (1.03-1.11)	<.001	1.09 (1.04-1.14)	<.001
Dyslipidemia	1.58 (0.94-2.65)	.08	1.76 (1.01-3.09)	.048
Lung cancer	1.69 (0.98-2.91)	.06	2.35 (1.20-4.57)	.01
Large intestine cancer	0.23 (0.06-0.93)	.04	0.45 (0.10-1.98)	.29
Blood cancer	0.31 (0.10-0.97)	.04	0.57 (0.14-2.37)	.44
Ovarian cancer	2.45 (1.31-4.57)	.005	2.82 (1.31-6.08)	.008
Pancreas cancer	3.60 (2.09-6.22)	<.001	4.24 (2.13-8.43)	<.001
Metastasis	2.21 (1.38-3.52)	<.001	1.70 (1.02-2.82)	.04

Crude and adjusted HRs and 95% CIs were estimated using subdistribution hazard models. We selected potential variables for the multivariate analysis including age, sex, and variables with *P* values <.10 on univariate analyses among baseline characteristics.

HR, hazard ratio.

In the sensitivity analysis using a stepwise method, no significant changes were observed in the relationships between the covariates and ischemic stroke events (Supplementary Table S2).

## 4 Discussion

The main findings of the current analysis were as follows: 1) the cumulative incidence of ischemic stroke was 4.0% at 1 year and 4.7% at 3 years among patients with CAVTE in the DOAC era; and 2) pancreatic cancer, ovarian cancer, lung cancer, dyslipidemia, metastasis, higher D-dimer at VTE diagnosis, and younger age were independent predictive risk factors of ischemic stroke in patients with CAVTE.

Several previous studies reported the incidence of arterial thrombosis including ischemic stroke after CAVTE diagnosis [[18], [19], [20]]. Brenner et al [20] reported a 1.1% incidence of arterial thrombosis among 5717 patients with CAVTE over a median follow-up period of 5.0 months, and Noumegni et al [18] reported a 6.2% incidence of arterial thrombosis in 914 patients with CAVTE over a mean follow-up period of 2.5 years, with both studies demonstrating a low rate of DOAC use. Larsen et al [19] focused exclusively on 298 patients with CAVTE receiving apixaban as DOAC, reporting a 4.4% incidence of ischemic stroke over 36 months. These findings suggest the relatively frequent onset of ischemic stroke after CAVTE diagnosis both in the pre-DOAC and DOAC eras. In line with the previous studies, the current study also showed that there were some ischemic stroke events in patients with CAVTE in the DOAC era (4.7% at 3 years). Moreover, the cumulative incidence of ischemic stroke was significantly higher in patients with metastasis than in those without (7.9% vs 3.4% at 3 years, respectively).

Investigation of risk factors of arterial thrombosis including ischemic stroke in patients with CAVTE has been limited. Two previous studies did not use multivariate analysis due to a small number of events [19,20], and another study, while employing multivariate analysis, included a low rate of DOAC usage [21]. Therefore, we conducted a comprehensive multivariable analysis to investigate risk factors of ischemic stroke in patients with CAVTE, which revealed several predictive risk factors including younger age, higher D-dimer at VTE diagnosis, and dyslipidemia as well as a specific cancer site and status. Among these findings, the cancer site was consistent with previous studies from cohorts of cancer patients as well as those of patients with CAVTE [19,[22], [23], [24], [25], [26], [27], [28], [29], [30]]. Moreover, the current study newly identified metastasis and elevated D-dimer at VTE diagnosis as independent risk factors for ischemic stroke, which have not been reported in CAVTE-specific research to date. These findings might help elucidate the mechanistic links between cancer and ischemic stroke, suggesting that metastasis of tumor cells via the bloodstream might promote the formation of microthrombi at the intravascular site, resulting in a hypercoagulable state [4,29,31]. This hypothesis could also explain the relatively low incidence of ischemic stroke from 1 to 3 years of follow-up. High-risk patients with metastasis may have been censored earlier due to cancer-related death, while the remaining cohort likely consisted of patients with early-stage cancer, who would have a lower risk of ischemic stroke. Additionally, the current study showed that only dyslipidemia was associated with an increased risk of ischemic stroke among traditional risk factors for ischemic stroke [18,[32], [33], [34]]. These results contrast with those from previous cohorts of patients with cancer, where diabetes, hypertension, and old age also significantly increased the risk of ischemic stroke [26,35,36]. The discrepancies might be partly due to differences in study populations between patients with cancer and those with CAVTE. Notably, the impact of age on ischemic stroke in the current study differed from the established association observed in general and cancer populations [26,36]. This finding could be attributed to greater hypercoagulability in CAVTE patients, making them prone to ischemic stroke, even at a younger age. Indeed, younger patients with ovarian and lung cancers might be associated with greater hypercoagulability than older patients with those cancer types (Supplementary Table S1). To our knowledge, no prior reports described a younger age as a risk factor in a CAVTE cohort, underscoring the need for further investigation.

In the ischemic stroke group, 25 patients (35%) were not receiving anticoagulation therapy at the onset of ischemic stroke. Considering the clinical importance of ischemic stroke after CAVTE diagnosis, appropriate risk assessment of ischemic stroke with careful follow-up may be crucial.

The current study had several limitations. First, it relied on an observational database, where treatment strategies, including the type, dose, and duration of anticoagulation therapy, were determined at the discretion of attending physicians, potentially influencing clinical events. Additionally, there is a potential risk of underreporting ischemic stroke events, particularly asymptomatic strokes or those with short symptom duration, which may have been missed based on the stroke definition used in this study. Second, we were unable to confirm medication adherence, which may have had some impact on the results. Third, the current analysis was conducted using data from a Japanese cohort, which may present differences in demographics, practice patterns, and clinical events compared with those from other regions. Thus, the generalizability of the current results should be carefully considered. Fourth, this study may have introduced a potential selection bias, as only patients with known active cancer at the time of VTE diagnosis were included, potentially excluding those diagnosed with cancer after the VTE diagnosis. Fifth, the absence of detailed information on the histologic type of cancer precluded analysis of the influence of cancer histology on ischemic stroke during follow-up. Sixth, the absence of detailed information on chemotherapeutic agents precluded analysis of their influence on ischemic stroke during follow-up. Seventh, the absence of detailed information, including the presence or absence of patent foramen ovale or right-to-left cardiac or pulmonary shunts, prevented us from determining the specific pathophysiology of ischemic stroke. Eighth, due in part to the small number of cases in cancer types other than those identified as risk factors in this study, it is possible that certain cancer types were not detected as significant risk factors for ischemic stroke. Ninth, the multivariate model may be overfitted due to the inclusion of 10 covariates compared with only 71 ischemic stroke events. However, we performed a sensitivity analysis using a stepwise method to refine the covariates, and no significant changes in the relationships were observed, further supporting the robustness of our results. Finally, patient characteristics, including D-dimer values, were obtained only at the time of VTE diagnosis and, so, may have changed over time.

## 5 Conclusion

In this large VTE registry in the DOAC era, the cumulative incidence of ischemic stroke was 4.0% at 1 year and 4.7% at 3 years in patients with CAVTE, and several independent predictive risk factors for ischemic stroke were identified, including pancreatic cancer, ovarian cancer, lung cancer, dyslipidemia, metastasis, higher D-dimer at VTE diagnosis, and younger age.

## APPENDICES

Kazuhisa Kaneda, Ryusuke Nishikawa, Yugo Yamashita; Kyoto, Japan. Ryuki Chatani, Kazunori Mushiake, Kazushige Kadota; Kurashiki, Japan. Yuji Nishimoto, Yukihito Sato; Amagasaki, Japan. Nobutaka Ikeda, Katsushi Amemiya, Masato Nakamura; Tokyo, Japan. Yohei Kobayashi, Ren Kimura, Tsukasa Inada; Osaka, Japan. Satoshi Ikeda, Yuki Ueno, Koji Maemura; Nagasaki, Japan. Kitae Kim, Ryo Shigeno, Yutaka Furukawa; Kobe, Japan. Moriaki inoko, Shinya Ito; Osaka, Japan. Toru Takase, Gaku Nakazawa; Osaka, Japan. Shuhei Tsuji, Mamoru Toyofuku; Wakayama, Japan. Maki Oi, Kazuaki Kaitani; Otsu, Japan. Takuma Takada, Kentaro Jujo, Nobuhisa Hagiwara; Tokyo, Japan. Kazunori Otsui, Kenta Mori; Kobe, Japan. Jiro Sakamoto, Toshihiro Tamura; Tenri, Japan. Yoshito Ogihara, Toru Sato, Kaoru Dohi; Tsu, Japan. Takeshi Inoue, Tetsuya Nadahama, Kunihiko Kosuga; Moriyama, Japan.Shunsuke Usami, Katsuhisa Ishii; Osaka, Japan. Po-Min Chen, Toshiaki Izumi; Osaka, Japan. Kiyonori Togi, Manabu Shirotani; Ikoma, Japan. Kazuhisa Kaneda, Takafumi Yokomatsu; Kyoto, Japan. Norimichi Koitabashi, Hideki Ishii; Maebashi, Japan. Seiichi Hiramori, Kenji Ando; Kokura, Japan. Kosuke Doi, Masaharu Akao; Kyoto, Japan. Hiroshi Mabuchi; Higashiomi, Japan. Yoshiaki Tsuyuki, Hiroto Yamamoto, Takeshi Aoyama; Shimada, Japan. Koichiro Murata, Eri Ishikawa, Ryuzo Nawada; Shizuoka, Japan. Kensuke Takabayashi, Mitsunori Miho, Shoji Kitaguchi, Takeshi Kimura; Hirakata, Japan. Hisato Nakai, Yuto Miura; Obama, Japan. Daisuke Sueta, Kenichi Tsujita; Kumamoto, Japan. Wataru Shioyama, Yoshihisa Nakagawa; Otsu, Japan. Tomohiro Dohke; Koka, Japan.
