# Supplementary material for: Incidence and risk factors of ischemic stroke in patients with cancer-associated venous thromboembolism: from the Contemporary Management and Outcomes in Patients With Venous Thromboembolism Registry-2
Source: Res Pract Thromb Haemost. 2024 Oct 30;8(8):102617. doi: 10.1016/j.rpth.2024.102617 (PMC11616038; doi:10.1016/j.rpth.2024.102617)
Supplement: Supplemental Material [file mmc1.docx]

**Supplementary Materials**

**e-Appendix 1: List of participating centers and investigators**

- Department of Cardiovascular Medicine, Graduate School of Medicine, Kyoto University, Kyoto, Japan: Kazuhisa Kaneda, Ryusuke Nishikawa, Yugo Yamashita (Kyoto University Hospital Ethics Committee; approval number: R3082)
- Department of Cardiovascular Medicine, Kurashiki Central Hospital, Kurashiki, Japan: Ryuki Chatani, Kazunori Mushiake, Kazushige Kadota (Kurashiki Central Hospital Ethics Committee; approval number: 3684)
- Department of Cardiology, Hyogo Prefectural Amagasaki General Medical Center, Amagasaki, Japan: Yuji Nishimoto, Yukihito Sato (Hyogo Prefectural Amagasaki General Medical Center Ethics Committee; approval number: 2021-3-50)
- Division of Cardiovascular Medicine, Toho University Ohashi Medical Center, Tokyo, Japan: Nobutaka Ikeda, Katsushi Amemiya, Masato Nakamura (Toho University Ohashi Medical Center Ethics Committee; approval number: H21053)
- Department of Cardiovascular Center, Osaka Red Cross Hospital, Osaka, Japan: Yohei Kobayashi, Ren Kimura, Tsukasa Inada (Osaka Red Cross Hospital Ethics Committee; approval number: J-0268)
- Department of Cardiovascular Medicine, Nagasaki University Graduate School of Biomedical Sciences, Nagasaki, Japan: Satoshi Ikeda, Yuki Ueno, Koji Maemura (Nagasaki University Ethics Committee; approval number: 21091305)
- Department of Cardiovascular Medicine, Kobe City Medical Center General Hospital, Kobe, Japan: Kitae Kim, Ryo Shigeno, Yutaka Furukawa (Kobe City Medical Center General Hospital Ethics Committee; approval number: 2021-09-17)
- Cardiovascular Center, The Tazuke Kofukai Medical Research Institute, Kitano Hospital, Osaka, Japan: Moriaki inoko, Shinya Ito (Kitano Hospital Ethics Committee; approval number: P211000100)
- Department of Cardiology, Kinki University Hospital, Osaka, Japan: Toru Takase, Gaku Nakazawa (Kinki University Ethics Committee; approval number: R03-170)
- Department of Cardiology, Japanese Red Cross Wakayama Medical Center, Wakayama, Japan: Shuhei Tsuji, Mamoru Toyofuku (Japanese Red Cross Wakayama Medical Center Ethics Committee; approval number: 2021-8-18)
- Department of Cardiology, Japanese Red Cross Otsu Hospital, Otsu, Japan: Maki Oi, Kazuaki Kaitani (Japanese Red Cross Otsu Hospital Ethics Committee; approval number: 650)
- Department of Cardiology, Tokyo Women's Medical University, Tokyo, Japan: Takuma Takada, Kentaro Jujo, Nobuhisa Hagiwara (Tokyo Women's Medical University Ethics Committee; approval number: 2021-0101)
- Department of General Internal Medicine, Kobe University Hospital, Kobe, Japan: Kazunori Otsui, Kenta Mori (Kobe University Hospital Ethics Committee; approval number: B210264)
- Department of Cardiology, Tenri Hospital, Tenri, Japan: Jiro Sakamoto, Toshihiro Tamura (Tenri Hospital Ethics Committee; approval number: 1228)
- Department of Cardiology and Nephrology, Mie University Graduate School of Medicine, Tsu, Japan: Yoshito Ogihara, Toru Sato, Kaoru Dohi (Mie University Hospital Ethics Committee; approval number: H2021-192)
- Department of Cardiology, Shiga General Hospital, Moriyama, Japan: Takeshi Inoue, Tetsuya Nadahama, Kunihiko Kosuga (Shiga General Hospital Ethics Committee; approval number: 20211119-02)
- Department of Cardiology, Kansai Electric Power Hospital, Osaka, Japan: Shunsuke Usami, Katsuhisa Ishii (Kansai Electric Power Hospital Ethics Committee; approval number: 21-067)
- Department of Cardiology, Osaka Saiseikai Noe Hospital, Osaka, Japan: Po-Min Chen, Toshiaki Izumi (Osaka Saiseikai Noe Hospital Ethics Committee; approval number: 20211103)
- Division of Cardiology, Nara Hospital, Kinki University Faculty of Medicine, Ikoma, Japan: Kiyonori Togi, Manabu Shirotani (Nara Hospital, Kinki University Faculty of Medicine Ethics Committee; approval number: 652)
- Department of Cardiology, Mitsubishi Kyoto Hospital, Kyoto, Japan: Kazuhisa Kaneda, Takafumi Yokomatsu (Mitsubishi Kyoto Hospital Ethics Committee; approval number: 21-28)
- Department of Cardiovascular Medicine, Gunma University Graduate School of Medicine, Maebashi, Japan: Norimichi Koitabashi, Hideki Ishii (Gunma University Hospital Ethics Committee; approval number: HS2021-138)
- Department of Cardiology, Kokura Memorial Hospital, Kokura, Japan: Seiichi Hiramori, Kenji Ando (Kokura Memorial Hospital Ethics Committee; approval number: 21082501)
- Department of Cardiology, National Hospital Organization Kyoto Medical Center, Kyoto, Japan: Kosuke Doi, Masaharu Akao (Kyoto Medical Center Ethics Committee; approval number: 21-034)
- Department of Cardiology, Koto Memorial Hospital, Higashiomi, Japan: Hiroshi Mabuchi (Koto Memorial Hospital Ethics Committee; approval number: 2021-05)
- Division of Cardiology, Shimada General Medical Center, Shimada, Japan: Yoshiaki Tsuyuki, Hiroto Yamamoto, Takeshi Aoyama (Shimada General Medical Center Ethics Committee; approval number: R3-11)
- Department of Cardiology, Shizuoka City Shizuoka Hospital, Shizuoka, Japan: Koichiro Murata, Eri Ishikawa, Ryuzo Nawada (Shizuoka City Shizuoka Hospital Ethics Committee; approval number: 2021-10-24)
- Department of Cardiology, Hirakata Kohsai Hospital, Hirakata, Japan: Kensuke Takabayashi, Mitsunori Miho, Shoji Kitaguchi, Takeshi Kimura (Hirakata Kohsai Hospital Ethics Committee; approval number: 2021-006)
- Department of Cardiovascular Medicine, Sugita Genpaku Memorial Obama Municipal Hospital, Obama, Japan: Hisato Nakai, Yuto Miura (Obama Municipal Hospital Ethics Committee; approval number: 2021-12-14)
- Department of Cardiovascular Medicine, Graduate School of Medical Sciences, Kumamoto University, Kumamoto, Japan: Daisuke Sueta, Kenichi Tsujita (Kumamoto University Hospital Ethics Committee; approval number: 2362)
- Department of Cardiovascular Medicine, Shiga University of Medical Science, Otsu, Japan: Wataru Shioyama, Yoshihisa Nakagawa (Shiga University Ethics Committee; approval number: R2021-094)
- Division of Cardiology, Kohka Public Hospital, Koka, Japan: Tomohiro Dohke (Kohka Public Hospital Ethics Committee; approval number: 2021-9-3)

**e-Appendix 2: The independent clinical event committee**

- Yuji Nishimoto, MD: Hyogo Prefectural Amagasaki General Medical Center.
- Kosuke Doi, MD: National Hospital Organization Kyoto Medical Center.
- Kensuke Takabayashi, MD: Hirakata Kohsai Hospital.
- Ryusuke Nishikawa: Graduate School of Medicine, Kyoto University.
- Kazuhisa Kaneda: Graduate School of Medicine, Kyoto University.
- Yugo Yamashita: Graduate School of Medicine, Kyoto University.

**e-Appendix 3: Definitions of patient characteristics**

Hypertension was diagnosed if peripheral blood pressure was >140/90 mmHg or the patient was taking medication for hypertension. Diabetes mellitus was diagnosed using hemoglobin A1c (HbA1c) [National Glycohemoglobin Standardization Program (NGSP), 6.5%] as the standard or was assumed if the patient was taking medication for the treatment of diabetes. Dyslipidemia was diagnosed if total cholesterol was >240 mg/dL, high-density lipoprotein cholesterol was <40 mg/dL, or the patient was taking statins. Chronic kidney disease was diagnosed if there was persistent proteinuria or the estimated glomerular filtration rate (eGFR) had been <60 mL/min/1.73 m^2^ for more than 3 months. The values of eGFR were calculated based on the equation reported by Japan Association of Chronic Kidney Disease Initiative [male: 194*Scr^−1.094^*age^−0.287^, female: 194*Scr^−1.094^*age^−0.287^*0.739]. Chronic heart disease was defined as persistent heart disorders including heart failure, history of myocardial infarction, and atrial fibrillation. Heart failure was diagnosed if the patient had a history of hospitalization for heart failure, the patient had symptoms due to heart failure [New York Heart Association (NYHA) functional class ≥2], or the left ventricular ejection fraction was <40%. Chronic lung disease was defined as persistent lung disorders such as asthma, chronic obstructive pulmonary disease, and restrictive lung diseases. Autoimmune disorder was defined as immune-mediated diseases, including inflammatory bowel disease, rheumatoid arthritis, and antiphospholipid syndrome [1]. History of major bleeding was diagnosed if the patient had a history of International Society of Thrombosis and Hemostasis (ISTH) major bleeding, which consisted of fatal bleeding, symptomatic bleeding in a critical area or organ, and bleeding causing a reduction in the hemoglobin level by at least 2 g/dL or leading to transfusion of at least 2 units of whole blood or red cells [2]. Hypoxemia was defined as arterial oxygen partial pressure of <60 mmHg or percentage saturation of hemoglobin with oxygen of <90%. Shock was defined as systolic blood pressure <90 mmHg for at least 15 minutes, a pressure drop of ≥ 40 mmHg for at least 15 minutes, or requiring inotropic support. Proximal deep vein thrombosis (DVT) in the lower extremities was defined as venous thrombosis located in popliteal, femoral, or iliac veins. Distal DVT in the lower extremities was defined as venous thrombosis located in calf veins including the peroneal, posterior tibial, anterior tibial, and soleus muscle veins below the knee. Anemia was defined as a hemoglobin level <13 g/dL for men and <12 g/dL for women according to the standard World Health Organization classification of anemia [3]. Thrombocytopenia was defined as a platelet count <100×10^9^/L [4]. Hereditary thrombophilia included protein C deficiency, protein S deficiency, and antithrombin III deficiency. Initial parenteral therapy included heparin (single or continuous injection), fondaparinux, and thrombolysis (urokinase or tissue plasminogen activator) within 10 days after the diagnosis. Antiplatelet drugs included aspirin, ticlopidine, clopidogrel, prasugrel, ticagrelor, and cilostazol.

**Supplementary Table 1. Median age of patients by cancer site and ischemic stroke occurrence**

|  | **Total**  **(N=1507)** | **Ischemic stroke**  **(N=71)** | **No ischemic stroke**  **(N=1436)** |
| --- | --- | --- | --- |
| **Cancer site** |  |  |  |
| Lung | 71 (65–77) | 69 (61–75) | 71 (66–77) |
| Blood | 70 (61–78) | 72 (66–75) | 70 (61–78) |
| Large intestine | 72 (64–79) | 76 (71–81) | 72 (64–77) |
| Uterus | 63 (56–70) | 61 (57–63) | 63 (56–71) |
| Pancreas | 70 (64–77) | 73 (62–78) | 70 (65–77) |
| Ovary | 60 (51–70) | 52 (44–58) | 60 (52–71) |
| Stomach | 71 (62–78) | 70 (67–72) | 71 (61–78) |
| Prostate | 78 (69–83) | 72 (64–79) | 78 (70–83) |
| Kidney/urinary tract | 72 (66–77) | 66 (66–66) | 72 (66–77) |
| Breast | 69 (60–77) | 75 (74–76) | 69 (60–77) |
| Gallbladder/bile duct | 73 (66–78) | 72 (68–72) | 73 (66–79) |
| Bladder | 73 (69–83) | – | 73 (69–83) |
| Brain | 64 (59–73) | 64 (64–64) | 64 (59–73) |
| Esophagus | 67 (62–73) | – | 67 (62–74) |
| Liver | 77 (72–81) | – | 77 (72–81) |
| Thyroid gland | 63 (61–70) | – | 63 (61–70) |
| Skin | 70 (61–78) | – | 70 (61–78) |
| Others | 71 (60–77) | 62 (58–71) | 71 (60–78) |

Age presented as the median and interquartile range.

**Supplementary Table 2. Risk factors for the development of ischemic stroke by subdistribution hazard model, applying stepwise selection based on the Akaike Information Criterion**

| **Variables** | **Crude HR**  **(95% CI)** | ***P*-value** | **Adjusted HR**  **(95% CI)** | ***P*-value** |
| --- | --- | --- | --- | --- |
| Age (each 10 years) | 0.86  (0.75–0.99) | 0.04 | 0.84  (0.71–0.99) | 0.04 |
| Women | 1.67  (1.02–2.74) | 0.04 | - | - |
| D-dimer (each 10 µg/mL) | 1.07  (1.03–1.11) | <0.001 | 1.08  (1.04–1.13) | <0.001 |
| Dyslipidemia | 1.58  (0.94–2.65) | 0.08 | 1.83  (1.05–3.18) | 0.03 |
| Lung cancer | 1.69  (0.98–2.91) | 0.06 | 2.73  (1.44–5.18) | 0.002 |
| Large intestine cancer | 0.23  (0.06–0.93) | 0.04 | - | - |
| Blood cancer | 0.31  (0.10–0.97) | 0.04 | - | - |
| Ovarian cancer | 2.45  (1.31–4.57) | 0.005 | 3.86  (1.95–7.65) | <0.001 |
| Pancreas cancer | 3.60  (2.09–6.22) | <0.001 | 5.08  (2.69–9.60) | <0.001 |
| Metastasis | 2.21  (1.38–3.52) | <0.001 | 1.74  (1.06–2.87) | 0.03 |

Crude and adjusted HRs and 95% CIs were estimated using subdistribution hazard models.

As a sensitivity analysis, we employed a stepwise method based on Akaike Information Criteria (AIC) to refine the initial 10 covariates, and then used the refined set of covariates in a subdistribution hazard model to reassess the relationships.

CI, confidence interval; HR, hazard ratio.

**Supplementary Figure**


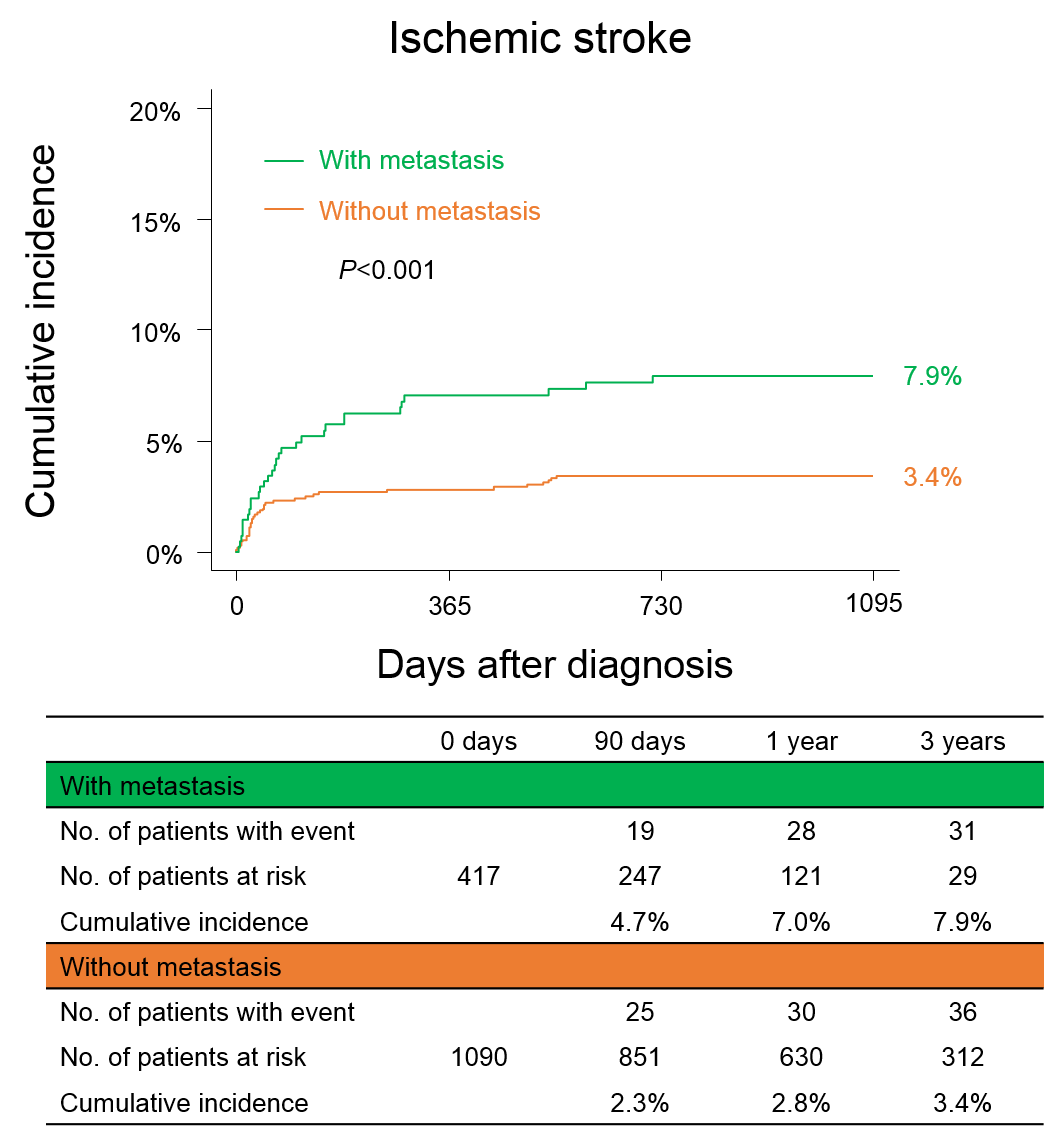


**Supplementary Figure.** Cumulative incidence of ischemic stroke in patients with metastasis and without.

The cumulative incidence function was used to estimate the cumulative incidence of ischemic stroke after VTE diagnosis.

VTE, venous thromboembolism.

**Supplementary References**

[1] Zoller B, Li X, Sundquist J, Sundquist K. Risk of pulmonary embolism in patients with autoimmune disorders: A nationwide follow-up study from sweden. *Lancet*. 2012;379:244-249

[2] Schulman S, Kearon C, Subcommittee on Control of Anticoagulation of the S, Standardization Committee of the International Society on T, Haemostasis. Definition of major bleeding in clinical investigations of antihemostatic medicinal products in non-surgical patients. *J Thromb Haemost*. 2005;3:692-694

[3] Nutritional anaemias. Report of a who scientific group. *World Health Organization technical report series*. 1968;405:5-37

[4] Yamashita Y, Morimoto T, Amano H, Takase T, Hiramori S, Kim K, et al. Influence of baseline platelet count on outcomes in patients with venous thromboembolism (from the command vte registry). *The American journal of cardiology*. 2018;122:2131-2141
